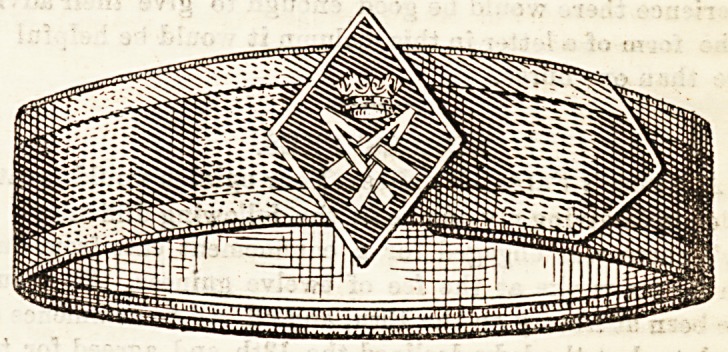# The Hospital Nursing Supplement

**Published:** 1895-08-03

**Authors:** 


### "The Hospital" Nursing Mirror.

Being the Extra Nursing Supplement of "The Hospital" Newspaper.

[Contributions for this Supplement should be addressed to the Editor, The Hospital, 428, Strand, London, W.C., and should have the word "Nursing" plainly written in left-hand top corner of the envelope.]

### News from the Nursing World.

### THE COLOURS OF OUR PRINCESS.

The armlet, which has now become the badge of the policyholders of the Royal National Pension Fund, has been issued under certain necessary restrictions. It must be returned when the holder ceases to be a member of the Fund, and whilst in her possession must neither be allowed to pass out of her hands nor be worn by any other person. On joining the Fund each policyholder will be entitled to receive an armlet from the secretary on depositing a shilling; a similar deposit having been made in the case of the nurses already in possession of this much admired badge.

### PERSONAL RECOGNITION.

"I had no idea that it would be so *personal*," said one of the policy-holders at Marlborough House. "Why the Prince and Princess spoke to ever so many, and talked particularly to those who happened to have nursed anybody known to them." The personal element, strongly marked through the whole proceedings of the memorable 26th of July, culminated in the graciously accorded interviews. "The Royal Family are much easier to get on with than some of the members of my committee!" was remarked by a guest, with an amused smile.

### HELP WELL BESTOWED.

Another nurse has written most gratefully of the rest which her fellow nurses have secured for her through the medium of The Hospital Convalescent Fund. "I already feel better," she says, "and I do hope that by the end of the month I shall be able to work again." Ill-health enforced a long and involuntary holiday on this writer, and absorbed most of her savings, and therefore a free bed in a suitable home has been a great boon to this tempoiarily incapacitated nurse. The Convalescent Fund was established with a view to retaining a permanent free nurse's bed at a seaside town, and the plan was carried out with satisfactory results. After a time appeals from suitable cases came to the hon. secretaries from all parts of the country, and proved the existence of drawbacks to the free bed being situated in the extreme south. Applicants for it could not pay the heavy fares or bear the fatigues of long journeys, whilst the fund was on far too modest a scale to provide for such expenses. It was therefore decided to make a fresh arrangement, and to send nurses to different health resorts, so that a place within a reasonable distance of each applicant might be available. This exceedingly practical arrangement has worked well, and will be carried out on a far larger scale when the fund is proportionately increased. All subscriptions will be promptly and gratefully acknowledged by the hon. secretaries of The Hospital Convalescent Fund, 428, Strand, London, to whom applications from nurses should be also addressed.

### "CAN'T AFFORD IT."

"I can't afford to join," is the regretful comment of many would-be policyholders; and it naturally gives rise to the question, "Why?" In the future it will probably be heard less frequently, as other institutions annually follow the example of the pioneer hospitals by assisting to pay a portion of the premiums for their nurses whilst their youth admits them at the lowest rate recognised by the Royal National Pension Fund. "I can't afford it" may therefore mean that an individual unassisted salary leaves no margin for investment, or it may express the inability of the speaker to "put by" for herself because her nearest relatives need a share of her earnings. But "I can't afford it" is sometimes the flimsy excuse of the nurse receiving high fees who has no one but herself to spend them on. In such cases it is inexcusable, and employers will soon cease to sympathise with the plaintive "I can't afford it" of the recipient of two or three guineas a week, whose earnings are absorbed between her cases in fine clothes unsuitable to her station, and in cabs, and in daily and hourly pleasure seeking. Truly the monotonous "I can't afford it" must be a plea accepted with reservation.

### GIRL LECTURES.

The various useful movements in which the National Health Society has from time to time distinguished itself are about to be counterbalanced by what seems to be a less commendable new departure. The society, in announcing that it is prepared to give preliminary training to would-be nurse-probationers, adds that these pupils can profitably fill up their time whilst waiting till they are old enough to begin their hospital training, by "earning money as lecturers!" Truly a pleasant prospect to girls anxious to refund themselves for their outlay for preliminary training; but will the propagation of such a type of lecturer be received with favour by any County Councils? These have done much of late in raising the status of health lecturers by selecting fully-trained experienced nurses for their work, and it is hardly likely that funds administered under the technical education schemes can consistently be diverted into the pockets of probationers in embryo. The society advertises itself prepared to provide lecturers at "varying charges" for county council work, on account of the increased number of lecturers they can command. Does this mean the encouragement of economy which must eventuate in the substitution of a "cheap girl" for the competent trained nurse-lecturer? How many of the former will be willing to take up the obscure life of a working probationer after assuming for two years the rôle of a competent teacher?

### A NOVEL FORECAST.

Imaginations of a high order appear to be owned by the two members of the Visiting Committee who recently made a report to the East Preston Board of

Guardians. They appear, from the statements in the Press, to have described in flattering terms the appearance of the nurses in their new caps and aprons, and also the gratitude of the recipients for these articles. It has since transpired that the nurses not having received their promised uniform, their improved appearance and proportionate gratitude were somewhat prematurely announced. The absence from the last meeting of the gentlemen whose fertile imaginations had originated the story prevented the explanation which the chairman was prepared to invite them to make.

### AN OPINION WORTH ATTENTION.

The Matron of Scarborough Workhouse has addressed to the guardians a letter which, on its publication in the local Press, must receive the consideration which it deserves. It calls special attention to the disadvantages under which the probationers labour as regards their training. "They have no nursing lectures—either medical, surgical, or midwifery, and are rarely even allowed to accompany the medical officer on his visits through the wards." The Matron (Miss C. Hodgson) is also strongly opposed to the plan for "so large a hospital to be left at night to the sole care of an inexperienced young probationer," and urges the engagement of a competent nurse about thirty-five for night duty. All Miss Hodgson's suggestions are worthy of attention, being the results of long experience. In concluding her letter, she remarks, "I feel assured it will be more economical, and your hospital will be more efficiently managed by appointing an experienced nurse than by increasing the staff of your probationers."

### PROGRESS AT KILMARNOCK.

The training provided for the present probationers at Kilmarnock Infirmary appears to be exceedingly thorough, and steps have been taken to give equal advantages of lectures, followed by examinations, to the nurses already established on the staff. Some of the latter have served the infirmary for periods of from three and a half to eight years, and during the last twelve months they have undergone three examinations, and received their diplomas. These, with the certificates gained by the probationers, were recently presented at a special meeting of directors, and the doctors added their testimony in a most gratifying manner to the excellence of the practical nursing carried on in the hospital wards.

### THOSE UNIFORMS AGAIN!

In spite of the protests of the leaders of the nursing profession, cordially and constantly endorsed by all earnest nurses, uniform continues an unsatisfactory adjunct to the calling of the trained nurse. The events of last week brought the subject again into prominence, and whilst "blouses" and "costumes" were exhibited by some who attended the meeting convened on 24th ult. at the small Queen's Hall, the large gathering on 26th ult., in the large hall, was exclusively attended by nurses in uniform. Washing materials seem now to be favoured by the majority of nurses, and with excellent effect, the style of many costumes furnishing a key to the kind of institute which produces them. "A good worker" may not infrequently be diagnosed by one glance at the cut and colour of her uniform. In contrast to cool-looking cotton dresses some of the thick woollen gowns are unsanitary and uncomfortable. If they do not show dirt, they are supposed to "save washing," according to the sentiments of a bygone age. The dresses of sisters and charge nurses have long been selected of washing materials at St. Thomas's and Charing Cross, the Temperance, and St. Bartholomew's hospitals, as well as at many others. The London Hospital sisters now have discarded their becoming navy gowns for commonplace pale-blue cottons. Navy-hued linens and other washing materials being procurable, it seems a pity such were not chosen for ladies in such responsible positions. Now they are no longer distinguishable in dress from scores of private nurses and masseuses!

### A GRATEFUL PATIENT.

Pleasant testimony to the unsectarian character of our large hospitals is given by a correspondent of the *Jewish Chronicle*. As the only Hebrew in a general ward, this patient has on various occasions passed months at a time in different hospitals, where her faith has been respected and her own disease skilfully treated. Her cordial appreciation of this consideration is shown in the words: "It is we who are strangers in a strange land, and should evince our gratitude daily for the many privileges we enjoy; things are far worse in other countries. So long as the doors of hospitals are not closed against us in sickness, so long as the doctors are kind and good, and so long as the nurses do their duty to suffering humanity, irrespective of creed, so long is a Jewish hospital not a necessity."

### THE BISHOP OF LONDON.

The annual meeting of the Hammersmith and Fulham District Nursing Association was very well attended, and the report showed a steady increase in its work. The chair was taken by the Bishop of London, who spoke of the association (which is affiliated with the Queen's Jubilee Institute) as supplementing the work of hospitals. He referred to the special value of nurses in carrying out the treatment ordered by the doctors. Poor people "were willing to take the medicine, but were often exceedingly unwilling to obey the doctor's orders, which were often quite as important as the medicine prescribed. Such a charity as this rendered a real service to the community. To restore a man to health was the greatest possible service they could render him." Further testimony to the value of the association, and earnest appeals for increased financial support were made by several other speakers.

### SHORT ITEMS.

The sum of £3 1s. 3d. has been contributed to the Burton Nursing Institution by seventy-six coopers, whose subscriptions are specially esteemed as they were unsolicited.—A pension of £26 per annum has been sanctioned by the Local Government Board for Sarah Collins, late nurse at Honiton Workhouse.—The Eton Board of Guardians have appointed a trained nurse to their workhouse.—The charge against a district nurse for having voted under the name of another nurse has been dismissed by the Altrincham magistrates.—A contemporary nurse has been appointed by the Keynsham Board of Guardians, who will assist the matron in the culinary and other details, reports the local press.—On the departure of Miss F. Lewis from Toxteth Infirmary she was presented with a handsome gold-mounted umbrella, as a token of the regard felt for her by the night nurses.—*Nursing Notes* for August contains an admirable article on "Thrift" by Miss Catherine J. Wood.—*The Daily Graphic* of July 27th and *The Lady's Pictorial* and *St. Paul's* of the current week contain illustrations of the R.N.P.F. nurses at the Queen's Hall.

### Elementary Anatomy and Surgery for Nurses.

By W. McAdam Eccles, M.B., M.S., F.R.C.S., Lecturer to Nurses, West London Hospital, &c.

### BURNS AND SCALDS.

Such injuries are of very frequent occurrence, and of special importance on account of their associated dangers. Burns and scalds are in their results much the same. A burn is that which is produced by actual flame, whereas a scald is the outcome of contact with steam, hot water, and other liquids. They vary as to their effects according to their extent, degree, situation, and the age of the patient. There are grave constitutional as well as local effects from burns and scalds. With regard to extent and situation, a burn on the trunk is more dangerous than a burn of corresponding superficial area on one of the extremities. It is generally said that when one-third of the skin of the body has been destroyed the case will be certain to end fatally. Burns and scalds are usually spoken of as being present in different degrees according to the depth of tissue involved; practically, however, there is no hard and fast line between the degrees, as they may be variously combined in the same burn. The degrees are six in number:—

The first degree is in reality the first stage of inflammation, and this is redness of the affected area, due to increased flow of blood through dilated vessels. Here there is no actual destruction of tissue, and consequently no scar will be left. Such hyperæmia of a small area is a trifling matter, but if the region injured be extensive, and the patient quite young, so much pain may be caused that what is called shock may be so great as to cause a fatal issue. It is manifest that such cases need stimulants and alleviation of the suffering as the method of treatment.

The second degree is present when the cuticle is raised and a blister is formed. Such a degree is the most common in scalds. Again, if extensive, this degree may be very serious. It is here as well as in the subsequent degrees that care in the removal of the clothing is important. The garments should on no account be pulled off, but gently lifted or cut off, so that no removal of cuticle may be occasioned. Exposure of the patient to cold air while the clothing is being removed must be always avoided. The burnt area is best covered with vaseline spread on antiseptic gauze or lint, any blisters which are tense being pricked to allow the serum to drain away, but the cuticle must not be removed. If there is much shock the patient must be kept warm, and stimulants given in small quantities, but frequently. Small doses of opium will relieve the pain, and thus tend to diminish shock. Some authorities are, however, strongly against its use.

The third degree is characterised by the true skin being only partially destroyed. The upper layers have been burnt and the deeper parts exposed. Very great pain is produced by the exposure and irritation of the cutaneous nerves.

The fourth degree is only a little deeper than the third, for in it the whole of the true skin is killed and the resulting pain will be but slight. In these two degrees the destruction of tissue will lead to scarring and deformity afterwards, but it will be infinitely greater after a burn of the fourth degree than after one of the third degree. The healing of these will always be tedious. The part actually destroyed by the burn constitutes what is termed a slough, and this has to be thrown off from the living tissues by a process of ulceration.

The fifth degree is constituted by the implication of the cellular tissue and muscles.

The sixth degree involves charring of the whole thickness of the limb.

After these four latter degrees certain stages, three in number follow, and require some explanation. The first is a stage of shock with usually much congestion of internal organs. Here the patient is pale, cold, or even actually shivering; the pulse is very feeble. At first there is intense pain, but it passes off and the result may be a state of coma or lethargy, which not infrequently ends in death. If recovered from, this stage will pass into the second, which consists of reaction and inflammation. The temperature rises, the pulse becomes rapid and bounding, and an area of redness will appear around the region of the injured part. In this stage there is a great tendency for inflammation of the lungs, the brain and its membranes, and the peritoneum and intestines, to supervene and carry off the patient. In the third stage suppuration is manifest, and generally begins about the fifth day after a burn. Great exhaustion may come on in this stage, and will require to be vigorously combatted.

Having thus briefly alluded to the conditions which generally follow burns, the precise treatment to be adopted must be stated. As has already been said, the clothes are to be carefully removed, and a dressing of gauze covered with vaseline, which may be warmed before its application, is to be used. The prevention of decomposition in the discharges is the end to be aimed at, but carbolic acid should not be used, as it is too irritating. When the sloughs have separated, dressing with boracic acid ointment answers well, and it is a good plan to change the application from time to time, say to zinc oxide ointment or to zinc oxide and calamine ointment. At this period again on no account must the dressings be allowed to become foul.

At a later stage when healing is slow, skin grafting may be employed. By this is meant the planting on the granulating surface of numerous portions of skin derived from another part of the patient's body. This skin should be removed so as not to cause any bleeding; and if a piece about the size of a threepenny piece has been excised it may be divided into many smaller pieces, and then placed on different spots on the surface. Over each piece a square of gutta percha tissue should be placed, and above all an absorbent antiseptic dressing. On about the fourth day the grafts will have taken if they are successful, and will appear now as little islets of transparent tissue. From these centres epithelium will spread, and in the end cover the part. During cicatrisation care must be taken to diminish the effects of the contraction of scar tissue by the proper use of splints and other apparatus.

### Notes and Queries.

### Queries.

(215) *Armlets.*—As one of the First Thousand I am most anxious to know where the armlet of the Danish colours is to be had?—*Nurse Anscombe.*

(216) *Stewardesses.*—Kindly tell me something about stewardess-nurses? —*Inglefield.*

(217) *Medical.*—Are there such things as hospital ships?—*Inglefield.*

(218) *Salary.*—Can you tell me where I could get a year's training in surgical work and receive a certificate and small salary in London?—*Nurse T.*

(219) *Massage.*—Please tell me of a useful book on this subject?—*Masseuse.*

### Answers.

215. *Armlet (Nurse Anscombe).*—Read Nursing Supplement of last week and this.

216. *Stewardesses (Inglefield).*—You must make application at the offices of the large shipping companies if you want such an appointment; you can find their addresses in the daily papers. If considered suitable your name will be put on their lists. A trained nurse takes the same position as the ordinary stewardess, making the beds of all lady passengers, &c., besides looking after the sick. Two hours daily off duty are granted and regular hours for meals. A nurse who can adapt herself to her surroundings and who is quick and orderly finds life on a good ship pleasant and healthy; there is generally plenty for her to do.

(217) *Medical (Inglefield).*—There are "hospital ships" at Long Reach where small-pox patients are nursed under the Metropolitan Asylums Board. In connection with the Mission to Deep Sea Fishermen there are hospital ships which accompany the fleets of fishing boats to the North Sea. This mission has also a beautiful hospital ship which goes to Labrador for six months every year, and takes doctors and stores for the fishers and their wives along the coast. Two English-trained nurses have accompanied this mission ship for three seasons. The offices of the Mission are at Bridge House, Blackfriars.

(218) *Salary (Nurse T.)*—Only as a paying probationer could you enter a general hospital for so short a time, but you might get experience of surgical work in a private nursing home. Certificates are of no value unless issued by an accredited training school. Why not make inquiries at some of the good provincial hospitals, and go in for three years' training?

(219) *Massage (Masseuse).*—"Art of Massage," by Mrs. Crichton Hale, published by the Scientific Press, 428, Strand.

### Royal National Pension Fund for Nurses.

### THE THIRD AND FOURTH THOUSAND.

### At the Queen's Hall.

It is nearly four years since the second thousand of the members of the Royal National Pension Fund for Nurses were received by the Princess of Wales at Marlborough House, and now, on Friday, July 26th, another and yet larger regiment were privileged to receive their certificates of membership from the same gracious hand. The Fund now numbers four thousand members, and of the third and fourth thousands over eight hundred gathered at the Queen's Hall, Langham Place, last Friday, to receive their invitations from Mrs. W. Burns, the wife of the chairman of the Council of the Pension Fund, to be instructed in the order in which they should approach the Princess, and to partake of lunch before being conveyed to Marlborough House.

To tell the truth, the resources of the Queen's Hall were rather severely taxed. The floor of the large hall was given up to the nurses' evolutions, while not only the small hall but the gallery were brought into requisition for the luncheon tables. At the first glance it seemed a difficult task to bring into order the crowd of excited women who were massed together in the area of the hall, but General Crease, C.B., who had undertaken to marshal them, has the habit of command, which means that he has firmness, patience, and good temper; and he was supported by experienced sergeants, who gathered the nurses into hundreds and ranged them four abreast. But, indeed, the General had every reason to be satisfied with his recruits. He had the advantage of dealing with women who have the habit of paying attention to what is said to them and of obeying orders. Even in this unfamiliar work their training told; they were quick, intelligent, obedient; and it may be that the General will compare unfavourably some masculine corps with the white-capped regiment he controlled last Friday.

When all were ready for the march Mrs. Walter Burns entered, accompanied by her daughter, and took up her place at the table on which lay the coveted invitation to Marlborough House, and as the nurses passed before her she shook hands with each as she presented her with her invitation. In a brief speech Mr. Burdett expressed to the nurses the gratification he felt at seeing them, and at Mrs. Burns's presence on the occasion. Mrs. Burns, as wife of the chairman of the Council of the Fund and daughter of Mr. Junius Morgan, whose generosity so largely helped to found it, has a special claim to the esteem of the nurses who belong to the Pension Fund, and has a special interest in them. In the present day, when the Fund is so securely established, even those who benefit by it can hardly recall the difficulty that was experienced in starting it, and the opposition with which it met. Therefore Mr. Burdett did well to tell the nurses how, when it was stated that as a preliminary to forming any such Fund £20,000 must be deposited with the Receiver in Chancery, Mr. Morgan offered himself to give the whole sum in order to establish the scheme. The generous donations of others made this unnecessary, but the gifts made by Mr. Morgan and his family to the Pension Fund show that the offer was no empty promise; and the presence of Mrs. Burns and her daughter prove also that the interest which Mr. Morgan felt in nurses is to be handed down as a sacred charge to his family.

After the presentation of the certificates came a new lesson to the nurses in the art and mystery of cheering. Women are not in the habit of expressing their emotions by calling out "Hip, hip, hip, hurrah!" at the top of their voices, and much laughter ensued when Mr. Burdett suggested that they should raise their voices in unison. But they responded gallantly, and if their cheer was set in a somewhat higher key than one generally hears, it was perhaps more melodious, and assuredly not less hearty. Mrs. Burns was cheered, and when Mr. Burdett had finished his speech a spontaneous and enthusiastic cheer arose.

Then the band of the Royal Artillery, which had been playing during the gathering and marshalling of the nurses, struck up again, and the heroines of the day went off to lunch.

Heroines!—and so they rightly wore a badge such as that we give to heroes. Her Royal Highness the President has lately instituted a badge which shall express her interest in the Royal National Pension Fund for Nurses, and attach them to her person. It takes the form of an armlet of red ribbon bordered with white, for red and white are not merely the colours of the nursing profession, but those of Denmark; they are the Princess of Wales's own, and therefore are they chosen for the badge which shall designate the nurses belonging to the Pension Fund. The ribbon is worn on the left arm, and ends in a red diamond-shaped fastening on which are embroidered her Royal Highness's coronet and monogram. This badge was worn last Friday for the first time, and will soon be in the possession of every nurse who is a member of the Pension Fund.

Omnibuses had been chartered to convey the nurses from the hall to Marlborough House. The large majority of them wore indoor uniform, with caps; only district nurses, who have no indoor head-gear, appeared in bonnets. But fortunately the sun shone, and no fear of drenching showers to "take the starch out of one" in mind or garments came to make them hesitate about occupying the outside as well as the inside of the conveyances. So with white streamers fluttering in the wind they drove down Regent Street, and many eyes turned to see the gay procession and note the bright, intelligent, happy faces.

### At Marlborough House.

At Marlborough House the nurses brigaded on the lawn under the shade of the elm trees, and there with the greater space at command their march was even more effective and accurate than in the Queen's Hall, and uniforms and badges could be more clearly seen. There was excitement, but there was no disorder when the Royal party appeared—the Prince and Princess of Wales, the Crown Prince of Denmark, Princesses Victoria and Maud, and the Duke and Duchess of Fife. The Duke of York joined the party at a subsequent stage of the proceedings. There were present also Lord and Lady Rothschild, Lord and Lady

Suffield, Mrs. J. Pierpoint Morgan, Mr. and Mrs. W. H. Burns, Mr. Walter Burns, jun., Miss Burns, Sir W. and Miss Broadbent, Mr. Thomas and Miss Bryant, Mrs. and the Misses Burdett (who received a spontaneous cheer from the nurses on their arrival), Dr. Hubert and Mrs. J. S. Bristowe, Mr. Herbert C. Gibbs, Mr. George Norman, Mr. and Mrs. Rawlings, Mr. George King, and the matrons of St. Thomas's, Guy's, Charing Cross, the Leeds General Infirmary, Royal Infirmary, Derby, Lewisham Infirmary, Miss Peter, of the Queen's Jubilee Nurses, and others.

One charming feature of the proceedings was the appearance of one of the little Ladies Duff, who stood by the Princess's side during the first part of the ceremony, and seemed greatly interested in the distribution, but after a time her little ladyship's interest in what must have seemed to her a mysterious business waned, and she withdrew.

The presentation of certificates passed off admirably with perfect order, but not without incidents which showed the quick eye of the Princess and the keen interest she takes in the nurses. Many badges were worn, and Her Royal Highness was quick to observe them all. She noted the badge of the Queen's Jubilee nurses, of whom there were many present, the Guild of St. Barnabas, the British Nurses' Association, the Nurses' Co-operation, and two nurses from the Seamen's Hospital, whose badge was, perhaps, the most interesting of all. It was a heart of red satin, with a ship in full sail embroidered on it, and it is a token of a sailor's gratitude, worked by fingers which have grown hard with handling ropes, to be given to the nurse who tended him in sickness. Uniforms, too, caught the Princess's eye—the picturesque red dress of the matron of a cottage hospital in Essex, and the practical fawn-coloured gowns worn by the nurses of the Lewisham Infirmary, whose costume also met with the approval of Lady Rothschild. A sensitive chord must have been touched when Her Royal Highness saw the nurse who had attended the Duke of Clarence, and she recognised also General Ponsonby's nurse. There was one Danish nurse in the band, and to her the Princess spoke in Danish, and she had a special word for a deaconess who had come from the Holy Land to be present.

### Speech by the Prince of Wales.

When all the certificates had been presented the nurses were bidden to draw near, and the Prince of Wales delivered the following address:—

Nurses,—It affords the Princess and myself much pleasure to receive you here to-day as the representatives of the third and fourth thousand who have joined the Royal National Pension Fund for Nurses. I understand that you come from all parts of England, Ireland, Scotland, and Wales. Our Fund is intended not only for nurses working within the United Kingdom but for all nurses throughout the British Empire. Several of the sisters of the Indian nursing service are amongst the warmest supporters of the Fund, and we now have members in every British Colony, a considerable number of them being at work in Australasia, at the Cape, and in Canada. I may remind you that the Fund is mutual and essentially co-operative, there being no shareholders or directors' fees, and therefore the whole of the profits go to the nurses. The Fund is open to every nurse, who may assure for a pension of any amount, commencing at any age, and for sickness and accidents also. In addition to the profit bonuses, there are the Donation Bonus Funds, which amount to upwards of £42,000, and the Junius S. Morgan Benevolent Fund, which has so far sufficed to relieve the necessities of every member requiring help, and to support twenty-five permanent pensioners, of whom fifteen are non-policy holders upwards of sixty years of age. Four years ago, when the Princess last had the pleasure of presenting certificates to the members, the invested funds amounted altogether to £100,000, and they now exceed £220,000. The premium income, which is entirely contributed by the nurses, is at present £35,000, and it is increasing on an average at the rate of £5,000 a year. The Fund is old enough to test the confidence which nurses have in it, and I find that the members are continually taking out additional policies, and that quite recently twelve nurses who had withdrawn have re-entered the Fund. The members have already withdrawn from the Fund no less than £17,000 of their savings. The reason given for withdrawal in the majority of instances is marriage, though a large number of nurses, when compelled to relinquish nursing for physical reasons, have been able to start on a new career owing to the investment of their savings in the Fund. The withdrawal of this £17,000 is an encouraging fact in itself, as the majority of nurses have declared that they would never have saved at all had it not been for the encouragement offered them by the Pension Fund. Since the Princess received the second thousand nurses the first quinquennial valuation by the consulting actuary, Mr. George King, has been made. It is satisfactory to find that in his report he states: "This, the first valuation of the Fund, proves conclusively that the guaranteed annuities will in future be materially increased out of profits. The Council and all the members are to be congratulated on the very prosperous condition of the Pension Fund disclosed by the valuation, and on the indication afforded of the large increase which will be possible in future in the annuities of those nurses who do not withdraw their membership." I am speaking not only as the patron of the Fund but on behalf of the Princess, your president, who has, from its conception, taken the greatest personal interest in the progress of this beneficent society. She has charged me say how delighted she is to receive you here to-day, and how much she hopes that the armlet in the Danish colours, suggested by herself in order to attach every nurse member of this Fund directly to her person, will serve to remind each member who wears it when she goes back to her work of ministering to the requirements and needs of the sick in any part of the British Empire, that the thoughts of the Princess are with every member of the Fund, and she hopes that this knowledge may increase their self-respect and stimulate them to greater exertions in the noble work to which they are devoting their lives.

Three hearty cheers were then given by the nurses for their Royal Highnesses the Prince and Princess of Wales.

### Mr. Burns' Reply.

Mr. Walter H. Burns, the Chairman of the Council, then said:—

May it please your Royal Highnesses,—It is my most agreeable privilege as chairman of the Royal National Pension Fund to tender to you the grateful thanks of the nurses for the unwearied kindness which you have always extended to them. For the third time we find ourselves in these beautiful gardens to receive from the gracious hands of the Princess, our president, the certificates which testify that the third and fourth thousand nurses have been accepted by her as members of the Pension Fund, and in addition thereto the beautiful badges which enrol them more especially as her nurses. Nothing could confer greater honour upon the nursing profession than the support which the Princess and yourself have extended to it. In the too often sad and trying scenes in the hospital wards and sick rooms amidst which their lives are passed, the nurses who are gathered here to-day will be able to cull and treasure some flowers of bright recollection from their gracious reception here to-day, and feel encouraged in their work by the thought that their labours are followed and appreciated by the highest personages in the land. But not only do I thank you for the nurses, it is also on behalf of the officers and directors of the Pension Fund that I desire to offer you our gratitude. None can know better than they how invaluable your sympathy and patronage have been to the cause. They have encouraged the nurses to join us and the public to support us, so that in less than six years, beginning with £20,000 and less than 500 nurses, we have now enrolled an army of between three and four thousand nurses, and an accumulated invested fund of over £200,000. I venture to say that no insurance or benefit society has ever been started which can point to such a record of success in so short a time; it is wholly due to the support which the Princess of Wales and you, sir, have given to our cause. Without you we should have done very little, but supported and encouraged by you we have been able easily to overcome all prejudice and all opposition. Your Royal Highness and the Princess have proverbially supported all charitable and useful works, but I venture to say you have extended your help to none which will do more good or reflect more credit upon your foresight than the Nurses' Pension Fund. However that may be, of one thing you may rest assured your sympathy and kindness are indelibly written on the hearts of your grateful nurses.

Great satisfaction was felt at the beauty of the certificates. "I thought they would only be a plain bit of printing," said one nurse, "and they are—lovely!" They were indeed exquisite, having been specially designed by Mr. Arthur Hacker, A.R.A. They show, appropriately, a beautiful figure of Sympathy, with outstretched wings and tender countenance, and the idea expressed in them will doubtless act as an inspiration to the nurses when the tension of their work seems to press them too sorely.

### After the Presentation.

Many of those present doubtless thought that when the formal proceedings were over the Royal party would withdraw, but, on the contrary, they stepped down and mingled with the nurses. Tea was served in a long tent, and the Princess herself conducted one of her guests to the table. The nurse so honoured was one of the older generation, who, when the Pension Fund was started was too old to join for such a pension as would keep her comfortably in her old age. But she joined for what she could, and with the growth of the Fund itself and the aid given by the Benevolent Fund, her pension will now reach the maximum amount. This case contains the moral of the Pension Fund—that it is a fund which helps those who help themselves. Open to all, it is friendly to all, and will provide for all, if only they show by their acts that they desire to raise a shelter for their old age. Of course, it cannot do anything for such nurses as the one who, although a private nurse in receipt of the highest pay, declared that she "could not afford" to join the Pension Fund. Can she afford, without its help, to provide for her old age? If so, how? And if not, would it not be better for her to economise a little now, in order to affiliate herself with an institution which exists in order to help nurses when they can no longer help themselves, and which gives with a generous hand to all who have done their best?

Not to one only did the Royal President and her daughters speak. They moved about among all, and few can have gone away without a gracious word, none without a friendly smile. At the same time, the President was the hostess, and truly made her guests feel "at home" within the Royal gates. The nurses stood about in groups or rested on the grass, enjoying the dainties provided for them, and listening to the strains of the Coldstream Guards, while the sun, which had mercifully kept behind the clouds during the earlier part of the proceedings, shone out to give additional charm and brightness to the ever-varying scene. Now one could observe better than when the nurses stood in closely-massed columns the various uniforms in all their details, and note with satisfaction how universally hospital authorities are beginning to recognise that uniforms may be plain and yet pretty. Of course, all are not equally attractive. Compared with the modern flat cap of starched lawn, the muslin caps of Derby Asylum seem unnecessarily high. But it was pleasant to notice on the breasts of its representatives the silver cross which showed that they had passed an examination in medico-psychology. Berrywood Asylum, which sent a solitary representative, has a handsome uniform of black cloth with a good deal of gold braid, which, however, though suitable for an asylum, would not do for the sick-room. It was, indeed, satisfactory to see that many hospitals are now clothing not only nurses but sisters in washing materials, evidently coming to the conclusion that there is no true dignity in dirt. Whole groups of nurses from one hospital might be seen. Miss Carvossa, the matron of the Royal Infirmary, Derby, brought a bevy of nurses, engaged a whole house for their lodging, and took them to the Indian Exhibition the night before the presentation. These Derby nurses will not forget their visit to London. But, indeed, none will. They have had courtesy, enjoyment, honour. They have been received by their Royal President, the fairest and most beloved of Princesses, in a way that shows that she is in no merely formal sense their head. They see that she works for them, that she cares for them, that she is, in the truest, broadest sense, their leader and their friend. And that knowledge will cheer and sustain them in their laborious life.

---

### WHAT A NURSE SAW AND HEARD.

### An Honoured Guest.

"Their Royal Highnesses won't forget that we are only probationers at ceremonial," remarked one of the oldest of the nurses, "and they must excuse a pro's attempt at a bow or a curtsey. I'm sure I don't know exactly which I made myself." But it was evident that no failure of respect was witnessed in the old lady's obeisance. She was seen later on taking tea under the personal superintendence of the Royal President of the Fund.

### What Was It?

"What will you take?" "Thank you, I've had some fruit." Then the first speaker asked another question. "But don't you want anything to drink?" "I've had that, too," rejoined her friend, "and very nice it was; I advise you to try it. To tell the truth," she continued, "I couldn't make out what it was. I never tasted anything like it. I'm sure it is not lemonade nor ginger beer, but it's refreshing." Her friend followed her advice, and then set down the pretty glass with a smile. "You dear old thing," she said laughing, "you've been enjoying champagne cup without knowing it!"

WORK OR PLAY.

"Oh yes, I'm certainly more tired than a day's hard work would have made me. It's partly the excitement, I suppose, but I'd gladly go through it all again if I could get another smile from that beautiful Princess."

AN INSPECTION.

"I thought it would be just like a school prize-giving, where one struggles through a crowd to receive something which is generally poked at one, and then one retires in undignified haste to make room for the next comer."

"That hardly describes the present scene," said her right hand neighbour, a refined-looking girl. "I've been watching Her Royal Highness, and I'm sure she *inspects* as well as *presents*. Nothing seems to escape her, and I believe she takes notice of everything that's not up to the mark," and the speaker tried to control a rebellious lock of curly hair before going to receive her own certificate. An approving glance fell for a second on the girl's neat figure and well-made cap, and she retired, feeling that she had passed muster, and, perhaps, was not considered unworthy to wear the distinguishing badge of the Royal President.

CAPS.

How the gusts of wind exposed the weak points of some of the caps! It lifted them up, and some half-dozen wearers at least faced the inspection with their caps in a perpendicular rather than a horizontal position. The Royal party seemed to enjoy these incidents thoroughly, and also smiled at the quaint nightcaps in which the Guy's Hospital probationers appeared disguised. On the heads of modern young ladies the shape lacked the appropriateness which marks it when placed on a grandmother's head.

ABSENCE REGRETTED.

"We feel that we must let you know," wrote two hospital sisters, "how sorry we are to be unable to receive our certificates from the hand of our much-loved Princess, whose kind interest in us and our work is a bright spot in many a dark hour." And other nurses kept away by duty thought longingly and lovingly of the bright scene in which they could not take part, except through their sympathy. Nurses are essentially "working women," and to snatch a whole day's pleasure is not possible for all. Eagerly did members of the First and Second Thousand encourage the Third and Fourth Thousand to be present. They knew by experience the pleasures of the former meetings, and cheerfully offered to do double duty rather than their friends should have to stay away. Many a private nurse working for herself cheerfully sacrificed a week's fees rather than risk being out of town on the longed-for day. Thus weeks of anticipation preceded the realisation of a pleasure which will form a precious memory in years to come.

IN THE STREETS.

When the nurses were perched within and without the omnibuses which carried them to Marlborough House, they were regarded with general interest along the route from Queen's Hall. "Hulloh! Where's 'ee off to?" asked a friend of one of the drivers from his seat on a passing omnibus. "Why, goin' to a tea drinking; and 'ee's that proud of his passengers 'ee carn't see 'is old friends to-day." The good-tempered chaff was interrupted by a policeman's voice, "Now then, young man, higher up, higher up! We shall have to buy you a bit o' freehold, and settle you there, if you can't move on a bit quicker." "Move on, ladies, move on, if you please," said another guardian of the peace when the nurses showed a tendency to linger in groups outside the garden entrance. "Move on, if you please."

When, at five o'clock, eight hundred capped and aproned figures emerged from the door, many decided it would be very nice to walk back to the Queen's Hall, where they had left their cloaks. For the next hour they were to be seen dotted about the West-end thoroughfares, distinguishable by the bright armlet, worn with conscious pride. "Are you too tired to walk back?" was asked, considerately. "Tired! No, indeed; I want to do all I can." And the two country nurses set off cheerfully to add another mile to those already accomplished that day.

### Our Princess's Colours.

THE armlet adopted on July 26th as the badge of the Royal National Pension Fund nurses is well depicted in the accompanying illustration. The ribbon is two inches wide with a centre stripe of rich crimson, and a white border is woven into each edge. The lining is of white ribbon over a piece of stiffened muslin, which makes as firm a foundation as a strip of cardboard would do. The band folds over, and is fastened with small hooks. The diamond-shaped shield is of crimson, outlined with a fine edge of white silk satin stitch, worked in white silk. The coronet surmounts the monogram of Her Royal Highness, and completes a badge which merits the cordial praise it evoked on all sides. The Princess of Wales approves highly of it, and her nurses adopt it with enthusiasm. In addition to the rules given below, it is well for all holders to realise that, although it may be arranged later on for a well-worn badge to be replaced by a new one, no nurse can, under any pretence, be allowed to retain two at the same time. It is expected that these rules shall be implicitly observed by all who have the privilege of wearing the Danish colours of Our Princess.

RULES FOR HOLDERS OF THE PRINCESS OF WALES'S ARMLET.

1. Every policyholder on joining the Fund will be entitled to receive an armlet approved by H.R.H. the Princess of Wales, upon depositing a sum of 1s. with the secretary of the Fund.

2. The Princess's armlet is to be returned to the Fund when the policyholder ceases to be a member, in which circumstances the deposit will be returnable on application.

3. The Princess's armlet is a *personal* decoration, identifying the holder as one of the Princess of Wales's nurses, and must in no case be allowed to pass out of her possession, or to be worn by anyone except herself.

4. The Princess's armlet is to be worn on the left arm, above the elbow.

5. The fact that the armlet has been issued to a member of the Pension Fund will be held to be evidence of her having undertaken to strictly carry out, and be bound by, the above conditions.—By order,

July 25th, 1895. LOUIS H. M. DICK, Secretary.

### Appointments.

THE COUNTY HOSPITAL, DURHAM.—Miss G. Eck has been appointed Matron of this hospital. She was trained at University College Hospital, Glasgow Maternity Hospital, and the London Hospital, and held the post of sister of the operation wards at Leicester Infirmary for several years. Miss Eck's nursing experience has covered a period of ten years, and she has held successively and with considerable success the posts of staff nurse, ward sister, and assistant matron. She has also taken matron's duties on two occasions, and has done private nursing and dispensing. Such varied experience forms excellent preparation for a matron's post, and we congratulate Miss Eck on her appointment. Many good wishes from her old friends will accompany her to her new work.

### Where to Go.

ROYAL AQUARIUM, WESTMINSTER.—Special performances on Bank Holiday commence at 10 a.m.

CHALFONT ST. PETER, BUCKS (station Chorley Wood).—The new home at the colony for the employment of epileptics will be opened by the Duke of Westminster on Wednesday, August 7th, at 4.45.

### Everybody's Opinion.

[Correspondence on all subjects is invited, but we cannot in any way be responsible for the opinions expressed by our correspondents. No communications can be entertained if the name and address of the correspondent is not given, or unless one side of the paper only be written on.]

### NURSING IN CAIRO.

"N. R." writes: Will any nurse kindly give me information as to the prospects of a nurse going to Cairo for the winter to take up private nursing? If anyone who has had personal experience there would be good enough to give their advice in the form of a letter in this column it would be helpful to more than one nurse.

### NURSES' FEES.

"A Nurse's Friend" writes: Your opinion would greatly guide me in giving my advice in the following difficulty. A lady's nurse was engaged for a confinement case from July 20th for six weeks at the fee of twelve guineas. She could have been at liberty on the 13th, and offered to commence on that date, but the lady declined the 13th and agreed for the 20th; whereupon the lady in the former case prolonged her engagement to the 20th. On June 27th (three weeks before the time) she received a note to say that the lady was unwell and that a temporary nurse was in the house, but that the doctor thought it might prove a false alarm, and saying she should hear again in the morning. Next day a letter arrived saying that the child was born and that her services would not now be required. She replied saying her engagement was from July 20th for six weeks, and that she was prepared to fulfil her engagement. No notice being taken of her letter, she wrote on the 18th to say that she should arrive on the 20th, when she was informed by letter that her services were not required, and referring her to the doctor, who would pay her what was fair, but saying if she had been disengaged on June 28th (which was three weeks before her engagement) this difficulty would not have arisen. The doctor offers half the fee. Of course, she has no other engagement for six weeks, and must return home or go into lodgings. What is customary under such circumstances?

[We believe the half-fee is all the nurse can claim.—Ed. *T. H.*]

### NURSING AT TOKIO.

Nurse Gertrude, nurse member of St. Hilda's, writes: I saw in your paper for May a short notice of the nursing work done at Kobé. May I venture to ask you to put into The Hospital a brief account of our nursing work in Tokio? St. Hilda's Mission is a community for general missionary work with a medical branch attached. This branch consists of free dispensaries and district nursing in Tokio. We have two dispensaries in different parts of the city. The larger one, in Azabu, quite near to St. Hilda's House, is opened on Monday, Wednesday, and Friday morning for two hours, when we have an average attendance of eighty persons (one hundred being the limit of attendances allowed at any one time), all of the poorest class. Our doctor is Japanese, also our dispenser. A matron and three Japanese nurses and an English one complete our staff. There is a small ward of three beds in this dispensary, so that we are able to take in for a longer or shorter period patients who are too ill to come each open day, or who are likely to be benefited by a few weeks' cleanliness and good food. Most of our in-patients are acute medical cases, so that here, as in larger hospitals, native nurses can be trained in district nursing. Our out-patients are chiefly slighter medical cases, with an average of twelve to fourteen surgical ones. On closed days our nurses go to visit these patients in their own homes to see that all who come are really poor, and also to see if they need any nursing; if so, such patients are nursed in their homes. This is extremely difficult sometimes, because Japanese ways are so totally different from ours, and consequently they do not always like to do as we wish, though, generally, they appreciate our methods when once they have tried them. Our other dispensary, in a district called Kiyobashi, is three miles from St. Hilda's, and is opened on Tuesday and Friday evenings for an hour and a half. The neighbourhood of Kiyobashi is densely populated and very unhealthy, for it consists chiefly of reclaimed land. Our work there is, from lack of workers, of necessity small. We do no district work, but confine ourselves to giving medicines and advice twice a week—our average attendance is twenty-five. It is impossible for one nurse to look after district nursing in more than one district. I think I can safely say that there is a greater opening in this neighbourhood for work than there was in Azabu, and in a very short time I feel sure the work would grow to the same extent; but it would be worse than useless to begin work that our present staff could not possibly carry on. I believe I am right in saying that ours are the only free dispensaries in Tokio, and patients from the most distant parts of the city come for advice and medicine. If we had one more English nurse we could open up the work in Kiyobashi at once and possibly start another simple dispensary in a fresh district. Will not some] lady who has had real experience in nursing, and who feels called to foreign missionary work in connection with our community, come out and help us, for we want help badly? Miss M. Bickersteth, The Palace, Exeter, is our secretary in England.

### CYCLING FOR NURSES.

Miss Florence Saunders, Lady Superintendent Peterborough District Nursing Association, writes: As the question of cycling for nurses has come up, I feel that I should like to say something on the subject. The Peterborough nurses were, I believe, the first to use machines for work, and began in the summer of 1893. At first we were a little in difficulty about dress, as evidently a long cloak could not be worn safely or conveniently. Finally we decided upon short capes of the same material as the Jubilee cloaks, with hoods for showery weather. The dark cotton Jubilee dresses and aprons of the same material look very quiet, and are not conspicuous. We wear elastic stirrups round the foot, which are buttoned on to the skirt to keep it down in windy weather. We have had a capital bag carrier devised for us by a friend, which is fastened on behind the seat, and does equally well for either the two or three-wheeled machine. With regard to the comparative usefulness of bicycle and tricycle, they both have advantages; the former is the lighter and cheaper, can be easily carried under shelter in showery weather, but the tricycle is easier to learn, and more comfortable in some ways. The saving of time in the week is enormous; our furthest district two miles off, is reached in eight or ten minutes without hurry. The great thing to impress upon the nurses is to ride quietly and not stoop over their machines. The windy weather is the greatest drawback and the heavy roads in spring, but I think there are few weeks in the year in which our machines are left in their stables. The bicycle has an advantage in there being only one track instead of three to think of. The weakest point in the dress of the cycling nurse in sunny weather is the bonnet; we must ask the Jubilee Inspector to allow us hats or devise something more sensible by next year. I am glad "An Old Nurse" has put away narrow-minded prejudice. Cycling can never be a graceful exercise for women, but it depends entirely upon the woman who practises it, be she either a "new" or an "old" one, whether it is objectionable or not. The most prejudiced people here acknowledge that we nurses have an excuse (born of common sense) for utilising cycles in the district, and Miss C. Wood, whose opinion everyone in the nursing world honours, congratulated me last year upon having taken cycles into use in spite of prejudice.

### Royal British Nurses' Association.

The annual meeting of the Royal British Nurses' Association was held on Wednesday, July 24th, at the Queen's Hall, Langham Place, W. Sir J. Crichton Browne, M.D., F.R.S., presided, and there were present amongst others: H.R.H. Princess Christian (president of the Association), Dr. Calvert (hon. treasurer), Dr. Bezley Thorne (hon. medical secretary), Dr. Alexander Davey, Mr. Mark Hovell, Mr. Pickering Pick, Mr. John Langton, F.R.C.S., Dr. R. F. C. Leith (Scottish hon. secretary), Miss Wedgwood (Royal Free Hospital), Miss Thorold (Middlesex Hospital), Miss Josephine de Pledge, and Miss Bunning.

The Chairman, in opening the proceedings, said: I have been requested by Her Royal Highness the President, who, I am sure, you are glad to welcome here to-day—(cheers)—to take the chair, and in the first place I will call on the secretary to read the notice calling this meeting. This having been done, four scrutineers were appointed to examine the voting lists for the election of the Council.

Dr. Bezley Thorne then presented the report, of which the following is an abstract:—

The history of the Royal Corporation of British Nurses since it last met in annual meeting, has been one of progress and development.

The bazaar at the Portman Rooms brought in about £600.

The lectures delivered under the auspices of the Association comprised two educational courses, the usual sessional lectures, and some delivered by Dr. Colman on the nursing of cases of nervous diseases.

The work of the Association continues to extend, and Miss Farquharson, the matron of the Alfred Hospital, Melbourne, has been appointed an honorary secretary of the Association in Melbourne.

In the month of December last a conference was held with the Irish members of the General Medical Council on the subject of the establishment of a National Branch in that kingdom. The project was met with unqualified approval, but it was suggested that some delay in its execution might be advisable.

Progress had been made in the design of establishing local centres of the Association in the provinces. The formation of a lending and reference library at the offices of the Association is being initiated, and it is expected that that useful institution will be in full operation by next October. The club-room and the arrangements for the provision of light refreshments in connection with it, continue to be regarded with increasing appreciation by members in London.

The opinions of three learned counsel have been obtained on the subject of retirement by rotation from the governing bodies of the Corporation. The opinion of the counsel of the Corporation is to the effect that that condition is obligatory on the members of the Council and of the Executive Committee. That of Mr. Swinfen Eady, Q.C., is that it is not applicable to the ex-officio members of the Executive Committee; while Sir Richard Webster expresses the view that, as regards the Executive Committee, the wording of the bye-laws is ambiguous. The three learned gentlemen are unanimous that, as regards the General Council, retirement is compulsory without exception.

The journal of the Association has been edited during the past year by Miss Josephine de Pledge with the success which is due to her literary ability and business capacity, and the thanks of the Corporation are due to that lady for the services which she has thus gratuitously contributed to the members at large.

Within the past year the membership of the Corporation has been diminished by the erasure of 156 nurses on account of their being more than two years in arrears in the payment of their annual subscriptions; while, on the other hand, the roll has been increased by the addition of 235 names. The withdrawals from the register have been seven in number, and the additions 282, all of whom fulfil the condition of membership of the Corporation.

The efforts of the Association during the coming year will in all probability be confined to the consolidation of its position, and to the extension of the work and projects already initiated, and especially to the establishment of local centres, to the task of revising and recasting the bye-laws, and to the extension of the Association in the sister kingdom of Scotland, under the direction of the hon. secretary, Dr. Robert Fraser Calder Leith.

The report was unanimously adopted.

Dr. Calvert in submitting the financial report, said: I am sorry I have to read these details because they are naturally uninteresting, and I think it would be a good thing in future if in the May issue of the journal of the Association the figures were printed, as then the annual meeting might take them as read. At the annual meeting held, very happily, at Windsor last year I stated that taking into consideration the increased expenditure due to the new office, together with the work of development which the executive committee had in hand, that it was very clear we must increase our annual income by about £300 a year. Since that meeting took place we have, in answer to an appeal made to the general council, added to our income in annual subscriptions for three years about £150 per annum, Her Royal Highness the President heading the list with an annual subscription of £25. (Cheers.) We also received £626 from the bazaar which, owing to the very active interest taken in it by Her Royal Highness, proved in every way a great success. Thus the finances of the Association have been placed for a time in a sound condition, but we are still, as we were last year, face to face with the problem of making our income in all respects equal to our expenditure. The legitimate way of doing this is, of course, by a large increase in the number of our members, and I am glad to say that in this respect we have made very considerable progress since the last annual meeting. During the year we have admitted 252 new members at 5s. each, which represents an increase in our annual income of £63. We registered 370, and if all of these had become members, the increase in our annual income would have been £93. May I again, as I did last year, beg of you all to do all you can to induce every nurse who registers to become a member of this Association? By doing so, you will materially assist in making your Association in a little time entirely self-supporting. I take this opportunity of assuring the members that we are as careful of the resources of the Association as we possibly can be. Without further remarks, I beg to move the adoption of this report. Mrs. Coster (St. George's Hospital): I beg to second the motion. The motion was then carried unanimously.

The Chairman: In order to put ourselves in order it may be desirable to have a resolution to carry out the suggestion which has been made by the treasurer. The elaborate, but hardly interesting, figures which he has submitted to you must convince you of the wisdom of the suggestion he has made that they should be printed in the number of the journal issued in the month preceding the date of holding the annual meeting. After a few words of explanation from the treasurer, this was agreed to.

Sir Spencer Wells then moved a vote of thanks to Her Royal Highness the president for her valuable services to the Association, and for her presence on that occasion. He said: I am quite sure everyone will feel indebted to her Royal Highness for the great interest she has taken in this Association from its very commencement, and only those who have watched the working of it, and have attended the meeting of the committee, can form any adequate idea of the continual care and attention she has displayed in its welfare. (Cheers.) The interest taken by our Royal president in the Association has been greatly to its advantage. (Cheers.) We have seen at times differences of opinion among members, but Her Royal Highness has always succeeded in smoothing away every difficulty and bringing about a state of harmony in the welfare of the Association. The nurses especially must feel this even more strongly than I can venture to express it, and therefore I will say nothing more, but content myself with moving a vote of thanks to Her Royal Highness for the interest she has shown at all times in the Association. (Cheers.) Dr. Buzzard: I have great pleasure in seconding the resolution.

The Chairman then put the resolution to the meeting, and after declaring it carried, amid loud cheers, he said: We have reason to know that the whole Association is thoroughly loyal and devoted to Her Royal Highness. (Cheers.) The general members of the Association do not know all that Her Royal Highness has been to this Association. Only those engaged in the management of it are aware of the amount of time, care, and attention our Royal president has bestowed on its affairs, and the generous and kindly support she is at all times willing to give it. I ask you again to signify your cordial acceptance of the resolution. (Cheers.)

Mr. J. Langton then said: It is my pleasant duty, your Royal Highness, Mr. Chairman, ladies, and gentlemen, to propose a vote of thanks to the honorary officers of this Association. It is one of the best traits of English life and character that people who have a large amount of work still condescend to undertake other work for the benefit of other people, and I am sure that our thanks are due to our honorary treasurer and to our two honorary secretaries for the amount of work which they do. (Cheers.) Only those brought into contact with them know the amount of detailed work which has to pass through their hands, as, practically speaking, I take it nearly all the work in its details has to be performed by them. For such labours I am sure this meeting will thank them most heartily and warmly. I, therefore, beg to propose, and I am sure it will be carried unanimously, a cordial vote of thanks to the honorary treasurer and the honorary secretaries for the admirable manner in which they have devoted themselves to the best interests of this Association. (Cheers.)

Miss Wedgwood (Royal Free Hospital): I have pleasure in seconding the motion. The motion was then agreed to unanimously.

Dr. Bezley Thorne, in acknowledging the motion, said: I am quite sure that I am only expressing the sentiments of my colleagues when I say we are deeply grateful to you for the handsome manner in which you have received this resolution. I need hardly say that it is a great pleasure to every one of us to work for the benefit of the Association, for we take the deepest interest in the welfare of nurses and in the advancement of nurses' training and teaching. It is therefore a great happiness to us to give what time we can spare from our professional duties to assist this great cause, for a very great cause it is. It is not only the better teaching and the better organisation of nurses that is our object, but also the raising of the vocation of nursing to the rank of a real profession, for we hope to see it brought to that position, and acknowledged by the public and the State as such. For these reasons we have the greatest pleasure in devoting all the time and energy we can to the welfare of this Association. We should singularly fail in our duty if we were not to give the Association our best energies when we have so brilliant and generous an example set us by our Royal president. (Cheers.) If anything could induce us with willingness and alacrity to bear the burden laid on us in the performance of our duties it would be the example the president has set us in all things. (Cheers.)

Mr. Pickering Pick: Your Royal Highness, Mr. Chairman, ladies and gentlemen, a very pleasant duty has been entrusted to me. It is to propose a vote of thanks to the Hon. Secretary of the Scottish Branch, Dr. Leith. This is, as we all know, the Royal British Nurses' Association, and we hope to establish a branch not only in Scotland, but in Ireland, Wales, and every one of the Colonies of Her Majesty's dominions. The success which has attended the establishment of the branch in Scotland encourages us to do so. But we are aware that the great success which has attended the Scot.ish branch has been due almost entirely to the indefatigable energy and zeal of Dr. Leith. (Cheers.) Miss Thorold: I will second it. The motion was carried, and Dr. Leith having replied,

The Chairman said: According to the report just handed me by the scrutineers, it appears that of the lists sent in 248 are unaltered, which is a clear majority, while 204 have alterations. The list, as recommended by the Council, will therefore be adopted with a few alterations, which the honorary medical secretary will now read.

Mr. Bezley Thorne then read the additional names, after which

The Chairman said: In consequence of some of those who have been proposed having found themselves unable to serve, I have to put before you a supplementary list to bring the number of matrons and nurses up to the number required, leaving a vacancy in each case, as it has been hitherto the custom to do, in order that the General Council may have power conferred on it by the annual meeting to elect members of the General Council in the course of the year, should any distinguished person join the Association whom it may be thought desirable to at once put on the Council. After reading the supplementary list the Chairman moved its adoption. Dr. Culvert: I will second it. The motion was then passed *nem. con.*

Dr. Bedford Fenwick, rising from the body of the hall, said: May I suggest, sir, that we have no power at an annual meeting to confer on the General Council power to alter their list? The General Council, by the charter and bye-laws, must be elected at the annual meeting. We are unable to give the Council power to elect itself.

The Chairman: In explanation of my proposal, I am informed that it has been the practice at every annual meeting since the formation of the Association for the annual meeting to confer on the General Council the power of adding to its members in the interim as vacancies may arise. I have the clearest recollection that such is the case, and that Dr. Fenwick proposed the same himself when secretary on a former occasion. If, on inquiry, it be found that the power is illegal, it will not be exercised.

Dr. Fenwick: I have to point out that when I proposed, as honorary secretary, what the chairman has just stated, this Association was not a corporation. It was not then acting under legal powers, and therefore we had a perfect right to do as we pleased. We are under legal powers now, and we must not act illegally.

The Chairman: The meeting may depend upon it that no irregularity of any kind will take place. Perhaps I may go further and ask this meeting to confirm the power asked for to appoint members of the Council during the year, provided that counsel's opinion is to the effect that that power rests in the Council. A motion to that effect was then put to the meeting, and declared to be carried.

H.R.H. Princess Christian: It is now my pleasure to move a vote of thanks to our chairman for his invariable kindness. He has taken the chair for me often and often, and he has done it far better than anyone else would have done. (Cheers.)

The Chairman: I can only thank you for the compliment you have just paid me, and I regard it as the crowning plume of my career as chairman, as it was proposed by Her Royal Highness. My duty to-day has been of a formal and exceedingly agreeable description, and I can only express the fervent hope and the firm belief that all occupants of this chair will have a similarly agreeable experience. (Cheers.)

The proceedings then terminated.

---

After the meeting in the Queen's Hall, the members of the association found their way to the St. James's Restaurant, where luncheon was prepared for them. Sir James Crichton Browne occupied the seat of honour at the head table, and there were also present the Rev. Dacre and Mrs. Craven, Dr. Bezley Thorne, Miss de Pledge, and Miss Alice Ravenhil (secretary to the association).

The nurses seemed thoroughly to enjoy themselves, and also to appreciate the amusing remarks with which Sir James Crichton Browne prefaced the toast of "The Queen." The health of the Princess Christian was then given, and cordially responded to. Dr. Bezley Thorne congratulated the Association upon its present flourishing position, and said that if he was not an admirer of the "New Woman" he certainly was of the "New Nurse," adding that as the science of the sick-room advanced nothing was more appreciated by medical men than the nurse of to-day, and that he looked forward confidently to her future recognition by the State. He trusted that the nurses of the Association would labour to remove certain misapprehensions which existed with regard to it among those who did not quite understand its aims.

A certain amount of annoyance was experienced by the nurses after the luncheon by the importunities of the waiters, who not only hovered behind each chair with a very evident eye to "tips," but where a nurse's intentions did not seem to be towards opening her purse-strings, they were not at all bashful in making their expectations known in a more unmistakeable manner. A nurse's funds are not as a rule very extensive, and as the lunch tickets were 2s. each, the further necessity of feeing the waiters really made the feast at St. James's Hall a rather expensive amusement. We feel sure this must have been unknown to the authorities, and is an abuse of which any repetition will be guarded against on future occasions.

Four o'clock saw the Queen's Hall once more besieged by the nurses, who, however, found Princess Christian there before them, and between then and six p.m. many of the members were personally presented to her Royal Highness, who pleased her audience immensely by herself giving them two solos on the piano. Certainly the nurses had reason to be grateful to the Princess for having devoted to them the whole of her day, and warm recognition of her kindness was heard on all sides. It was a pity that some uniformity of dress was not *de rigueur*, or that some nurses cannot be taught a sense of propriety in the matter of clothes; for the wonderful blouses, not to mention hats and, above all, fringes, which were to be seen inside and outside the Queen's Hall on Wednesday, the 24th, were a really terrible reflection upon their wearers.

The Princess drove off just after six o'clock, and the nurses rapidly dispersed homewards after a pleasant day's entertainment.

## Figures and Tables

**Figure f1:**